# The Implementation of Shared Decision-Making Using Patient Decision Aid Tools to Select Breast Cancer Treatment Options: A Systematic Review in the Time of Minimum Quality Standards

**DOI:** 10.3390/healthcare13070748

**Published:** 2025-03-27

**Authors:** Olatz Lopez-Fernandez, Carmen P. Aguilar Castillo, Bárbara Horrillo, María Luisa Sánchez de Molina Ramperez, Héctor Guadalajara

**Affiliations:** 1 Department of Behavioural Sciences Methodology, Faculty of Psychology, Universidad Nacional de Educación a Distancia, Moncloa-Aravaca, 28040 Madrid, Spain; 2 Instituto de Investigación Sanitaria Fundación Jiménez Díaz, 28040 Madrid, Spain; carmen.aguilarc@quironsalud.es; 3 Psychiatry Department, Hospital Universitario Fundación Jiménez Díaz, 28040 Madrid, Spain; 4 Faculty of Health Sciences, Universidad Villanueva, 28034 Madrid, Spain; bhorrillo@cescisneros.es; 5 Centro de Enseñanza Superior Cardenal Cisneros, 28006 Madrid, Spain; 6 Department of General and Digestive Surgery, Hospital Universitario Fundación Jiménez Díaz, 28040 Madrid, Spain; mluisa.sanchez@quironsalud.es (M.L.S.d.M.R.); h.guadalajara@quironsalud.es (H.G.); 7 Anatomy, Histology and Neuroscience Department, Faculty of Medicine, Universidad Autónoma de Madrid, 28029 Madrid, Spain; 8 Surgery Department, Faculty of Medicine, Universidad Autónoma de Madrid, 28029 Madrid, Spain

**Keywords:** shared decision making, patient decision aid, breast cancer, treatments, education, healthcare

## Abstract

**Background**: Research on shared decision making (SDM) has significantly increased in the 21st century. This study aims to review publications that include patient decision aid (PtDA) tools for selecting medical treatments for breast cancer (BC) since the advent of the minimum International Patient Decision Aid Standards (IPDAS) quality criteria. **Methods**: A systematic review was conducted using the PRISMA statement and focused on the literature published between 2013 and 2024. The databases included PubMed, Google Scholar, and PsycINFO. The quality of the studies was critically assessed. **Results**: A total of 29 empirical studies were examined, involving research conducted in Europe, America, and Asia. Most of the studies were quantitative clinical experiments, although qualitative and mixed methods were also reviewed. Three key themes were extracted: (1) study characteristics, including countries, sample sizes, and methodologies; (2) the clinical characterises and outcomes of the SDM processes and the implementation of PtDA tools; and (3) the various versions of the IPDAS criteria utilised. **Conclusions**: The medical option currently proposed includes a range of treatments, both surgical and nonsurgical options. Evidence shows positive outcomes associated with this healthcare approach; however, only half of the studies assessed utilised tools that met IPDAS criteria. Challenges remain in integrating SDM and PtDA tools into routine clinical practice, yet risk factors and potential solutions have been identified.

## 1. Introduction

Shared decision making (SDM) is a practice where clinicians and patients collaborate to make informed decisions by sharing the present scientific available evidence [1,2]. This healthcare approach supports patients in considering their clinical options while ensuring their preferences are communicated to the clinicians [3] (e.g., if the patient prioritises maintaining her breast, the surgeon should be aware to consider treatments that align with this). It is essential for patients to acquire a comprehension of their health conditions, treatment options, and the associated risks and benefits. This understanding promotes patients to properly articulate their values and priorities when communicating with clinicians. The employment of decision-making tools into SDM can enhance communication skills to foster a more collaborative healthcare context.

In the case of SDM in oncology, patient decision aid (PtDA) tools are being developed and tested to support the SDM process, but it is uncertain whether these devices increase the use of SDM by providers effectively [4]. On one hand, a pre-pandemic study [5] showed a mere 36% of the patients and surgeons perceived their consultation as SDM-based. Interestingly, it was observed that surgeons perceived the SDM more frequently than patients did but with scarce objective measures (e.g., validated metrics). On the other hand, a post-pandemic study [6] examined the criteria for SDM in oncology, emphasising the involvement of decision makers (patients and clinicians), decision-specific criteria (e.g., IPDAS), and contextual factors (e.g., hospital). Leinweber et al. in 2019 [7] identified 107 PtDAs to help oncologic patients to make informed decisions, but only 39 were developed to assist patients in selecting treatments and 5 intended to aid in the selection between a major open surgical procedure and a less invasive option. Furthermore, the majority of tools employed were designed to enhance patient decision making in breast cancer (BC). As one of the most prevalent oncologic diseases worldwide, BC ranked as the second highest in incidence according to the World Health Organization [8]. These factors significantly informed the selection of BC as the focus of the present study.

To the best of the authors’ knowledge, only a few systematic reviews have targeted the role of PtDA in BC care. Zdenkowski et al. [9] identified 23 PtDA tools between 2011 and 2015 for early BC care, in which decisions addressed included the choice between breast-conserving surgery and mastectomy, the administration of chemotherapy and/or endocrine therapy, radiotherapy, and fertility preservation. The outcome measures were heterogeneous, with most studies reporting a reduction in decisional conflict and an increase in both knowledge and satisfaction among patients, with no changes in levels of anxiety or depression. Spronk et al. [10] identified seven oncologic PtDA tools between 2006 and 2021, of which three were designed for metastatic BC. These tools showed applicability across various stages of the healthcare process and were useful in facilitating decisions regarding care, independently of the chemotherapy.

Indeed, the International Patient Decision Aid Standards (IPDAS) Collaboration initiative was established in 2003 to enhance the quality of healthcare in SDM. The framework focuses on the components of the PtDA tools that support informed, values-based reasoning and engagement with healthcare professionals [11]. There are six IPDAS versions of the criteria: the original with 80 items within 12 broad criteria [12], the IPDAS Checklist (74 items [13]), the short version IPDAi Assessment (47 items [14]), the minimal criteria proposal IPDAS Checklist (44 items [15]), the Standards for Universal reporting of patient Decision Aid Evaluation studies (SUNDAE) Checklist (26 items [16]), and, lastly, the IPDAS 2.0. Checklist (11 core domains [17]).

This systematic review seeks to provide a comprehensive and updated analysis of clinical studies related to SDM within the context of BC medical treatment. It specifically focuses on the utilisation of the PtDA tools to evaluate their characteristics and effects in clinical practice. The specific aims are the four following: (1) to examine the type of studies conducted in this area of clinical healthcare practice; (2) to assess the PtDA tool main characteristics and the medical treatment options they present; (3) to evaluate the main and secondary outcomes achieved; and (4) to identify, where applicable, the version of the IPDAS utilised in the development of the PtDA tool used for selecting BC treatments through SDM.

## 2. Materials and Methods

Guidance from the Preferred Reporting Items for Systematic Reviews and Meta-analyses (PRISMA) statement [18] informed the methods for conducting and reporting the present comprehensive systematic review.

### 2.1. Search Strategy

The following scientific bibliographic databases were searched: PubMed, PsycINFO, and Google Scholar. The review process was undertaken with an equation as follows: (((“breast cancer”) AND (“treatment*” OR “therapy*”)) AND (“shared decision-making” OR “SDM”) OR (“decision aids” OR “patient decision aid” OR “PDA” OR “PtDA” OR “decision aid tool*” OR “decision aid app*”)) NOT “screening” NOT “diagnostic”. Additionally, the Google Scholar database, only titles were selected with the equation as follows: allintitle: (“shared decision making” OR “SDM” OR “app” OR “PtDA”) AND (“breast cancer”).

### 2.2. Eligibility Criteria

The systematic review was initiated in 2013 with the official launch of the minimum quality standards for the implementation of PtDA tools 2.0. by the IPDAS [15].

The studies included in the present review were selected based on predetermined inclusion criteria: (1) it assessed SDM concerning the selection of medical treatments following a BC diagnosis, using a PtDA tool in an SDM clinical context; (2) it constituted an empirical study (i.e., employing quantitative, qualitative, or mixed method approaches); (3) it was published during the period selected (2013–2024); (4) the articles were retrievable as a full text within a peer-reviewed journal; and (5) the language used was English, Spanish, or French. The exclusion criteria were as follows: (1) studies that assessed SDM in other oncological pathologies not targeted (e.g., prostate cancer), with no inclusion of the BC, or that addressed nonmedical treatment options (e.g., screening methods); (2) papers that were theoretical research papers (e.g., reviews and meta-analyses or protocols); (3) publications that were outside the selected period (e.g., prior to 2013 or 2025); (4) articles that could not be retrieved electronically or were not published in peer-reviewed journals; and (5) languages used were other than those specified.

**Figure 1:**
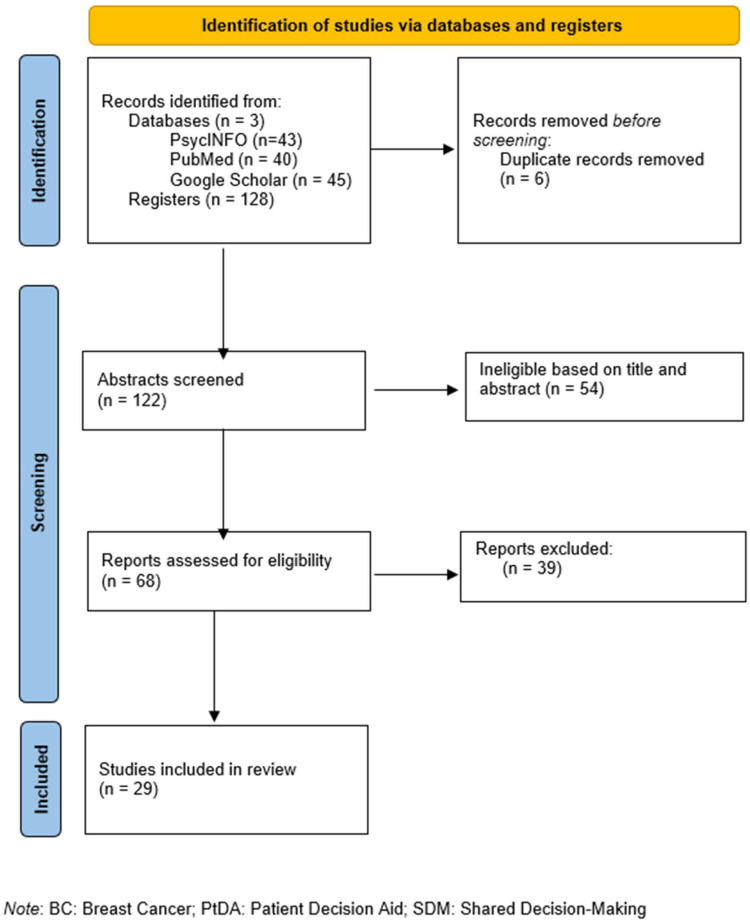
Flow diagram following PRISMA guidelines [18].

A total of 128 papers were initially identified from the three selected databases. Following the removal of duplicate entries (*n* = 6), 122 publications were screened based on their titles and abstracts. From this group, 54 studies were excluded for not meeting the predefined criteria (e.g., systematic reviews and conference abstracts). This resulted in 68 reports that underwent further evaluation through a comprehensive review of the retrieved articles. Of these, 39 studies were excluded due to various reasons (e.g., the absence of a clear application of SDM and PtDA tools or nonmedical treatment options). Consequently, a total of 29 studies were identified for consideration in this evaluation. (see Figure 1).

### 2.3. Selection Process

The selection of studies was conducted through a rigorous three-stage process. Initially, three reviewers applied the search strategy by utilising the defined equation within a database to identify the initial set of publications. The second stage involved a comprehensive review to remove duplicates and ensure that each selected study adhered to the aims (C.P.A.C., O.L.-F.). This phase included a careful evaluation of the titles and abstracts from the primary pool of articles. Following discussions and consensus reached during meetings, another refined pool of papers was established. In the final stage, the full texts were meticulously screened by two reviewers, comparing their content against the inclusion criteria through two rounds of evaluation. The first round aimed to identify clear publications that corresponded with the designed aims (C.P.A.C., B.H.). The second round was undertaken in cases where the inclusion of an article was uncertain, in which it was reviewed by a secondary reviewer (O.L.-F.). A collaborative discussion was conducted to reach a consensus on whether to include or exclude the article from the final selection.

Figure 1 depicts the study selection process in accordance with PRISMA guidelines [18]. A total of 29 studies were included in the current review after the aforementioned criteria.

### 2.4. Quality Study, Data Extraction, and Management

The quality and risk of bias were assessed at a study level using a critical assessment tool selected from the Joanna Briggs Institute (JBI)—Critical appraisal tools—in concrete, the Checklist for analytical cross-sectional studies (Moola et al., 2015 [19,20]). This choice was made because all the research designs examined were cross-sectional in nature. The studies were analysed according to Moola et al.’s eight questions [20]. Differences in results were resolved through discussion (C.P.A.C., O.L.-F.).

All the papers that appeared to meet the inclusion criteria were qualitatively assessed using the full text according to a set of predefined themes. The key pieces of information were extracted, including authors and year of publication (with country), aims and available BC treatment options, sample characteristics, study design, and measures employed. Additionally, the PtDA tools’ characteristics applied within an SDM process were noted, along with main study results with SDM outcomes, as well as the IPDAS version applied, when appropriate. A thematic synthesis was conducted, with all authors actively participating in the data extraction. The BC surgeon led the extraction of medical treatment options and outcomes when relevant (M.L.S.-R.), which was supervised by the senior surgeon (H.G.), while health psychologists and a methodologist provided insights on other variables (C.P.A.C., B.H., O.L.-F.), including the versions of IPDAS identified (B.H., O.L.-F.).

## 3. Results

A total of 29 studies meeting the inclusion criteria have been summarised in Table 1. These studies employed clinical samples and examined medical treatment characteristics for BC with a focus on SDM facilitated by a PtDA tool. The analysis revealed three primary themes: (1) study characteristics, including countries, sample sizes, and methodologies; (2) the clinical characteristics and outcomes of the SDM processes and the implementation of PtDA tools for BC medical treatment selection; and (3) the various versions of the IPDAS utilised. The forthcoming results section will provide a brief detailed discussion of each of these themes following Table 1.

### 3.1. Study Quality Results

To evaluate the methodological quality of the studies (see Table 2), the Checklist for analytical cross-sectional studies [19] was undertaken. The eight questions of the assessment were addressed, with the exception concerning confounding factors in a few studies [37,38,39,40]. Additionally, as this Checklist was applied to the qualitative or mixed methods studies [47,48,49], a few questions were considered either nonapplicable or partially applicable, respectively (e.g., question 8 about statistical analysis), as in similar healthcare studies [50].

### 3.2. Key Methodological Features of the Selected Studies

Among the 29 studies, almost half of them (*n* = 13) were conducted in Europe, with contributions from the Netherlands [21,32,39,44,47,48], Germany [31,36], the United Kingdom [43,46], Denmark [38,40], and Switzerland [35], followed by a third from North America (*n* = 10) [23,24,25,26,27,28,29,33,34,45]. Lastly, a fifth of the articles (*n* = 6) were conducted in Asia, including Taiwan [37,41,42], China [22,49], and Japan [30].

The sample sizes across these studies ranged from 10 [47] to 1339 participants [46]. In 58,6% of the studies (*n* = 17; [22,23,24,25,27,29,30,33,34,35,36,40,42,43,46,48,49]), the samples comprised only patients, while 10,3% (*n* = 3; [21,39,44]) included both patients and clinicians (usually physicians). Additionally, 20.7% of the studies (*n* = 6; [28,31,38,41,45,47]) featured patients along with a variety of other healthcare professionals (e.g., nurses), while only one study [32] focused on physicians and other providers.

In the domain of research designs, a hierarchy of strategies is typically observed, organised from those exhibiting the greatest to the least internal control. The following outlines the designs: (1) Randomised Controlled Trials (RCTs) [22,24,30,33,34,35,36,43,45,46], (2) Factorial Experimental Designs [23], (3) Quasi-Experimental Designs [41,44], (4) Mixed Methods Designs [27,31,37], (5) Qualitative Designs [21,30,39,47,48,49], and (6) Observational Designs [29,38].

In the context of research techniques, questionnaires emerged as the principal instrument for data collection. At the baseline phase of the SDM study, several validated SDM tests were employed, such as the Decisional Conflict Scale (DCS [22,26,30,35,41,42,44]), particularly its subscale on decision making [22,24,30], the Decision Making Styles Inventory (DMI [25]), Preparation for SDM (PrepDM [27]), the nine-item Shared Decision-Making Questionnaire (SDM-Q-9 [44])), the Decision Satisfaction Scale (DSS [27]), and the Control Preferences Scale (CPS [44]). Following decision making, the post-test phase usually comprised the Decision Regret Scale (DRS [22,40,42,46]), the Observing Patient Involvement Scale (OPTION [42]), the Subjective Decision Quality scale (SDQ [33]), the Control Preferences Scale (CPS [34]), the SDM Process Scale (SDMPS [34]), and the collaboRATE tool [44,46].

Additionally, oncological measures were incorporated, including BC-specific questionnaires, such as the Body Image Scale (BIS [42]) and a range of quality-of-life questionnaires developed by the European Organisation for the Research and Treatment of Cancer (EORTC), such as the Cancer Breast, with a 23-item cancer-specific supplement (QLQ-BR-23 [46]).

Finally, selected studies integrated psychological assessments to evaluate the impact of the PtDA tools on the mental health of BC patients. These assessments included validated scales for pre-surgical and post-surgical anxiety, such as the Spielberger Short State-Trait Anxiety Inventory (STAI [30,46]), and the Hospital Anxiety and Depression Scale (HADS [22,26,45]), alongside coping strategies measured by the Brief COPE inventory [46].

### 3.3. Clinical Characteristics and Outcomes of Decision Support Tools for Breast Cancer Treatment

The majority of the studies identified primarily focus on early BC [22,23,24,25,26,27,28,30,32,40] apart from locoregional BC [33,46,47], including fertility considerations [21,35]. The medical treatment options identified (by frequency order) were:

(1) Breast-conserving therapy (with radiotherapy) or mastectomy (with or without radiotherapy) [23,24,30,32,39];
(2) Mastectomy or breast-conservation therapy [33,34,37,45];
(3) Surgery and decide whether to use adjuvant therapy or not (e.g., hemotherapy, human epidermal growth factor receptor 2 targeted treatment, endocrine treatment, zoledronic acid treatment, and/or adjuvant radiotherapy) [38,40,46,47];
(4) Breast reconstruction surgery options (e.g., implant-based breast reconstruction or autologous) [41,42,48];
(5) Surgery options, breast-conserving therapy and radiation, and mastectomy (with or without reconstruction) [25,49];
(6) Cryopreservation of embryos, ovarian tissue, or none [21,35];
(7) Surgery plus breast reconstruction, breast-conserving therapy, mastectomy, or mastectomy with breast reconstruction [22,29];
(8) Mastectomy (with reconstruction or not) or breast-conserving therapy with radiation [27,28];
(9) Breast-conserving therapy with radiation, mastectomy, watchful waiting, and breast-conserving surgery without radiation [31,36];
(10) Mastectomy or not [26];
(11) Primary endocrine therapy or surgery with adjuvant therapies or to have adjuvant chemotherapy after surgery or not [43];
(12) Radiotherapy or not [44].

In terms of the types of PtDA examined, these include a variety of formats, such as leaflets and paper-based booklets [16], multimedia resources (e.g., videos with animations [21], websites [34], option grids [27,28], visual tools that present various treatment options alongside their associated risks and benefits [23,34], mobile applications [42], patient narrative tools [23,25], and value clarification exercises [21,27,30]).

The clinical studies on BC treatment options yielded significant outcomes based on the classification by Légaré et al. [4]. The main outcomes identified were:

(1.1) Shared decision making [28,30,31,32,33,34,35,36,38,39,40,41,42,43,44,45,46,47,48,49];

(1.2) Costs and cost analysis [36,40,45];

(1.3) Decision making [24,25,26,27,29];

(1.4) Observer-reported outcome [28,34,38,39,43,47,48];

(1.5) Health-related quality of life [37,41];

(1.6) Patient-reported outcome [21,22,37,42,45].

The secondary outcomes identified were:

(2.1) Patient outcomes

- Affective-cognitive outcomes [21,22,24,25,26,27,29,35,37,41,42,44,46,47];
- Knowledge [21,22,24,26,27,28,29,30,41,44,46,47,48];
- Satisfaction [24,27,28,34,38,41,42,45,48];
- Decisional conflict [22,24,26,29,30,35,41,44];
- Decision regret [22,24,30];
- Patient–clinician communication [36,45];
- Self-efficacy [29,47];
- Empowerment [25,26].

(2.2) Behavioural outcomes

- Match between preferred option and decision made [27,36,41,42,47].

(2.3) Health outcomes

- Health-related quality of life [47];
- Anxiety [29,37];
- Depression [22,24,37];
- Stress [29,37].

(2.4.) Process outcomes

- Consultation length [23,36,45].

Regarding the usages of the PtDA tools, research indicates that these could play a significant role in mitigating conflicts in decision making among patients, particularly in the context of choosing between lumpectomy and mastectomy [26,30,34]. These tools are usually associated with improvements in patient knowledge, enhanced participation in the decision-making process, and increased satisfaction with the decision-making experience (e.g., [30]). Nonetheless, the effectiveness of PtDA tools in improving information perception is not consistently observed. A study evaluating a web-based PtDA for breast reconstruction following mastectomy [34] found that it did not enhance the patients’ perception of the conveyed information when compared to high-quality websites. However, this study did report an increase in satisfaction.

Additionally, patient narratives, whether presented in a text or video format, could substantially influence information-seeking behaviours in PtDA tools. A study discovered that patients who viewed video narratives engaged more with information concerning mastectomy, while those who read text narratives were more inclined to focus on lumpectomy information [23]. Furthermore, the inclusion of patient narratives in PtDA tools may reduce reliance on anecdotal reasoning and facilitate a clearer understanding of the risk–benefit ratios associated with various treatment options, thereby enhancing SDM [24,25]. Illustrative materials and comics may also serve as effective tools within PtDA, particularly for patients with low health literacy [28,37,45]. Indeed, providing training for physicians in SDM practices may alleviate anxiety and depression in cancer patients [22].

Therefore, the implementation of PtDA tools within clinical practice presents a variety of challenges in the BC treatment [43]. Risk factors include practical barriers, insufficient professional commitment, resistance to altering established practices, and constraints related to time. Conversely, protective factors may include support from management, effective integration with electronic health records, and thorough training for staff [28,36]. The creation of PtDA tools in collaboration between patients and clinicians has the potential to enhance their relevance and acceptance among users [47].

The PtDA tools ought to be developed and employed in an ethical manner. It seems essential to address disparities in health literacy and numeracy to ensure that PtDA tools are accessible and comprehensible for all patients [25,26,28]. Numerous articles [21,37] discuss critical considerations for the design and content of PtDA tools: the simplification of medical jargon, the enhancement of navigational features, the provision of clear explanations, and the incorporation of visual aids, such as comics, can significantly increase the accessibility and understandability of PtDA tools.

### 3.4. IPDAS Applied in the Decision Support Tools for Breast Cancer Treatment

Regarding the use of the different versions of the IPDAS criteria, almost half of the papers (*n* = 14) informed about using a specific version for the PtDA tool. Interestingly, the SUNDAE Checklist was not indicated by any article, and the last IPDAS 2.0. only was cited in an article. Additionally, a few papers cited the IPDAS versions without explicitly mentioning if a version was used [22,30].

- 2005 IPDAS (80 criteria/items in 12 broad criteria) [29,30];
- 2006 IPDAS Checklist (74/64 items) [21,27,30,32,44];
- 2009 IPDAi Assessment (47 items) [28,31,36,38,48];
- 2013 IPDAS Checklist (44 items) [40,49];
- 2021 IPDAS 2.0. Checklist (11 core domains) [47].

## 4. Discussion

This systematic review study was designed with four specific aims: (1) to examine research related to SDM in BC using PtDA tools; (2) to evaluate the influence of PtDA tools on decision makers in BC treatment selection; (3) to assess the healthcare outcomes achieved through their use; and (4) to identify how the IPDAS versions are utilised.

In examining the studies published in this specific clinical practice between 2013 and 2024, it appears that SDM is more prevalent in Western cultures, leading in the European continent (central and north Europe), followed by the USA and Asia. Similar systematic reviews [9,10] have identified comparable regions, noting that the USA [9] and the Netherlands [10] are the countries with higher prevalence rates of SDM in early and metastatic BC, respectively. Another systematic review found the primary global regions publishing clinical practice guidelines (CPGs) and consensus statements (CSs) about SDM in BC are precisely Europe and North America [51]. The studies included in the present review were assessed to be of high-quality level according to the JBI Checklist for the Cross-sectional Studies, similar to the findings of other comparable reviews [9,10].

In terms of research methods, the RCTs and other manipulative designs are the more common strategies. Nevertheless, nonmanipulative methods, such as mixed methods, qualitative, and observational studies are also present. Previous similar reviews identified a balance of methods between RCT and other designs [9,10], which highlights that the field is evolving towards more controlled and comprehensive SDM in BC studies. This development also probably explains why standardised and validated tests are typically applied to collect evidence on the changes produced through the PtDA use in relation to SDM styles, perceptions regarding BC, and the psychological issues associated with the diagnosis. Indeed, contrary to the findings of de Mik et al., 2018 [5], a significant number of objective measures were applied in the quantitative studies analysed. The SDM tests commonly utilised include a set applied in pretest measures (e.g., DCS and DMI) and another set employed in post-test measures (e.g., DRS and Option). Recent studies have begun to incorporate tests focused on concerns related to BC (e.g., EORTC scales), as well as anxiety tests before and after applying the SDM process (e.g., HADS and STAI).

Regarding the role of the decision makers, the present findings indicate that only a minority of studies include both patients and clinicians as participants, while a larger number of publications involve only patients. Other systematic reviews focusing on SDM in BC have shown that PtDA tools do not effectively promote their actual use in clinical practice (e.g., tools should be integrated in the clinical workflow [27]). Concerning the options provided, most SDM interventions have been designed to reflect the specific medical treatment options (e.g., whether undergoing mastectomy or pursuing adjuvant therapies), compared to patients’ attributes (e.g., like age and literacy levels). This highlights the imbalance between the clinical factors and personal attributes considered in the PtDA tools and decision-supported interventions studied [52]. The limited inclusion of physicians (e.g., surgeons and oncologists) may indirectly contribute to biased perception of the need for SDM [10]. There is also a need for SDM training that is tailored to various clinical roles, both within and outside consultations (e.g., coaching and on-the-job instructions for healthcare providers [36,39]). Furthermore, existing PtDA tools need clear instructions on how and when to use them, with strategies to enhance communication between clinicians and patients to determine the best treatment option based on the clinical and personal factors.

The present review identified fewer PtDA tools for BC compared to the review undertaken by Leinweber et al. in 2019 [7]. However, those identified (e.g., BRAID, iCanDecide, and Pink) were developed in various formats to assist patients in selecting BC treatments, including invasive surgical procedures and less invasive options. As noted by Zdenkowski et al. [9], the decisions evaluated included the choice between breast-conserving therapy and mastectomy, whether to undergo surgery and apply adjuvant therapy, radiotherapy options, and considerations for fertility preservation. In the present findings, before discussing radiation options, breast reconstruction emerged as a crucial shared decision to make. Other treatment options included watchful waiting (especially in frail patients) or breast-conserving surgery, among others. Additionally, a recent review emphasised surgical decision making in BC treatment, covering mastectomy, breast-conserving therapy, unilateral mastectomy, contralateral prophylactic mastectomy, and breast reconstruction decision [53]. This indicates that our review, along with that recent one, encompasses a broader range of BC treatment options than similar previous reviews [9,10].

The main outcome measures [4] highlighted in this systematic review indicate that SDM and observed patient-reported outcomes are particularly prevalent in the present findings. This suggests a promising link between the artificial intelligence and the patient-reported outcomes regarding the BC treatment options. It is worth noting that some relevant results were excluded from this review because they were based on protocols from ongoing RCT (e.g., Cinderella project founded by the European Union [53]). In terms of secondary outcomes, improvements were observed in both patient and health-related areas, cognitive-emotional aspects (i.e., anxiety and depression) and decisional conflict decreased, while knowledge and satisfaction increased [9]. From a behavioural perspective, the alignment between the patient’s personal preference and treatment options emerged as the most significant factor; however, the time spent in consultation has been identified as a key obstacle. SDM in BC seems to face challenges at various levels. At the system level, while decisions rights and autonomy are generally upheld in modern societies, the implementation of personalised healthcare remains in its infancy [54]. At the individual level, patient-related factors (e.g., psychology and education), clinician-related factors (e.g., attitudes and consultation styles), and the interaction between the two (e.g., doctor–patient trust and communication) influence SDM. Additionally, decision-making behaviours (e.g., encompassing information, patient preferences, and use of the PtDA) could facilitate SDM or lead to passive decision making, the latter being more prevalent at BC healthcare [54,55].

Concerning the existing versions of the IPDAS, the most commonly used criteria include the IPDA Checklist with 74 items, combined through 64 items [13], and the IPDAi Assessment [14], each representing 17% of the total. However, half of the studies reviewed assessed the quality of the PtDA tools. A systematic review focused on evaluating the IPDAS for early-stage BC treatment from 2006 to 2018 [56] found a wide variation in the quality levels of the PtDA tools, which showed limited adherence to the IPDAS Checklists. Notably, the communicative aspects were identified as the weakest. It could be inferred that utilising available multilanguage resources and providing online training on IPDAS Checklists, among other options (e.g., CPG and CS), could improve adherence to best practices when applying IPDAS criteria to PtDA tools for BC decision making.

## 5. Conclusions

The open debate on SDM in the clinical field [57] still requires further investment in the systemic factors. A number of well-designed studies utilising a diverse range of methods have been reviewed, revealing a set of heterogenous PtDA tools for SDM in BC treatment options. These medical interventions expand the available options from the past, including nearly all available treatments across surgical and nonsurgical options with an evolution of the technologies used. Positive general and specific outcomes show beneficial results with this healthcare approach. However, only half of the studies included tools assessed with an IPDAS criteria, and explicit information about the number of criteria or scores achieved is often lacking. It is noteworthy that, despite the promising results of these tools, there are challenges in integrating them into routine clinical practice. The implementation of educational technologies and personally reported outcomes, combined with the clinical outcomes, appears essential to effectively engage decision makers during and outside the clinical encounters and within the workflow of the oncologic treatment process, which could involve multiple stages.

## Figures and Tables

**Table 1:** Clinical studies reviewed.

Author, Year [Reference] (Country)	Aim and Treatment Options	Sample	Design	Measures	PtDA Characteristics	Results and Outcomes	IPDAS
Garverlink et al., 2013 [21] (The Netherlands)	To improve information about fertility preservation for BC patients. Options: Cryopreservation of embryos, ovarian tissue, or none	*N* = 185 Participants S2: *n* = 10 Patients S3: *n* = 8 less educated women, *n* = 140 healthy students S4: *n* = 17 Clinicians *n* = 10 patients	Development in 4 stages: 1. Draft 2. Acceptability 3. Knowledge in healthy population 4. Acceptability revised PtDA in patients and physicians	S2: structured interviews. S3: knowledge tests. S4: Likert scales about layout and content.	Web-based PtDA with values clarification. Medical content consists of 5 chapters with 26 pages. PtDA contains values clarification exercise and a question prompt sheet.	PtDA regarded as a relevant source of information PtDA seemed coherent and understandable. Outcomes: Patient outcomes: affective-cognitive outcomes: knowledge	2006 IPDAS Checklist (64 items). 5/48 criteria with regard to the content and development process of DAs could not be met.
Lam et al., 2013 [22] (China)	To explore the effectiveness of a PtDA beyond consultations Options: Early BC surgery and, when applicable, breast reconstruction, breast-conserving therapy, mastectomy, or mastectomy with breast reconstruction	*N* = 276 T1: *n* = 138 IG take-home booklet *n* = 138 CG standard information T2: *n* = 237 *n* = 118 IG *n* = 119 CG T3: *n* = 216 *n* = 110 IG *n* = 106 CG T4: *n* = 214 *n* = 107 IG *n* = 107 CG	Randomised controlled trial.	Interview-based questionnaires at 4T after consultation: Primary outcomes: - 1 week after (T1): DCS, decision-making difficulties, BC knowledge - 1 month after (T2): Decision Regret Secondary outcomes: Treatment decision, Decision Regret, postsurgical anxiety and depression at - 4 months after (T3) - 10 months after (T4)	After a pilot study, the PtDA results in a booklet with content based on current clinical guidelines for surgical management of early-stage BC. The PtDA booklet comprises four components: 1. Differences among treatment options. 2. Review of benefits and disadvantages. 3. Personal values clarification worksheet. 4. Overview guidance and suggested next steps.	Primary outcomes: IG had lower DCS. No differences in Decision-Making difficulties, BC knowledge nor Decision Regret. Secondary outcomes: IG lower Decision Regret; T4: IG scored less in HADS-Depression. No differences at HADS-Anxiety scores, HADS- Depression at T3 nor treatment decision. Outcomes: Patient outcomes: affective cognitive outcomes: knowledge, decisional conflict, decisional regret. Health outcomes: depression,	Based on IPDAS with no score.
Shaffer et al., 2013 [23] (USA)	To examine the impact of video and text-based narratives on information search in a Web-based patient PtDA for early-stage BC. Additional aims: To distinguish between the effect of narratives and the effect of videos. Options: Breast-conserving therapy with radiotherapy or mastectomy	*N* = 56 Women *n* = 36 Video: Video narrative IG Video control CG; *n* = 20 Text: Text narrative IG Text control CG	Multilevel modelling.	Two text versions of the Web PtDA by replacing the patient and physician interviews with text transcripts of the videos	Participants had access to video controls that allowed them to play, pause, and move to different positions in the video timeline. PtDA included videos of physicians providing didactic information about early-stage BC and the treatments.	Participants viewing PtDA with patient narratives spent more time searching for information than those with PtDA without it. Narratives appear to have a global effect on information search. Outcomes: Process outcomes consultation length	
Shaffer et al., 2013 [24] (USA)	To examine the effect of patient narratives that discuss decision processes versus patient experiences on decisions about treatments for early-stage BC Options: Breast-Conservatory Therapy with radiotherapy or mastectomy	*N* = 302 Women	2 (content: process versus experience) × 2 (evaluative valence: positive only versus mixed) factorial design.	Information. Search task - 9 DM process Likert scale Women in narrative conditions also completed: 4 Connection with narratives (influence, emotionality, and helpfulness)	MouselabWEB uses an interactive table to display info. and track info. search. The table presented info. about the 2 treatment options and different decision dimensions: General info., radiation treatment, breast reconstruction, Surgical details: Length of hospital stay, Discomfort, Recovery, Side effects, Appearance, Local recurrence	Participants viewing process narratives spent more time searching for info. Participants viewing experience narratives reported a greater ability to imagine treatment’s experience; they also evaluated their decision more positively on several dimensions. Outcomes: Patient outcomes: affective cognitive outcomes: knowledge, decisional conflict, decisional regret, satisfaction Health outcomes: depression Decision making	
Shaffer et al., 2014 [25] (USA)	To evaluate the effect of narratives used in a popular, public PtDA on hypothetical treatment decisions and attitudes toward the PtDA and explore the moderating effects of participant numeracy, electronic health literacy, and decision-making style. Options: early-stage BC surgery, lumpectomy and radiation, mastectomy with or without reconstructions	*N* = 200 Women *n* = 100 IG stories from BC survivors *n* = 100 CG no stories from BC survivors	Randomised controlled trial.	eHEALS electronic health literacy. Decision-Making Styles Inventory: decision styles. Subjective Numeracy Scale: numeracy. Likert-scale items: confidence decisional difficulty, likelihood of changing their mind, and feeling overwhelmed. Overall quality, perceived helpfulness, satisfaction, likelihood of recommending the video, emotionality, trustworthiness and credibility	Narrative video PtDA made with large video about surgical options for early-stage BC and short section of another video about breast reconstruction. The result was a narrative video, 1 h long, and included stories from 12 BC survivors. The narratives covered 3 topics: patient’s emotional reaction to the diagnosis, strategies for DM (subscale of the DCS), and discussions about the aspects of the surgeries. The control video was created by removing the patient stories.	Narratives affected motivations for treatment decisions and perceptions of the aid’s trustworthiness and emotionality. Narratives had no effect on preferences for surgical treatments or evaluations of the PtDA quality. Outcomes: Decision making Patient outcome: affective-cognitive outcome: empowerment	
Manne et al., 2015 [26] (USA)	To test the acceptability and preliminary efficacy of a novel interactive web-based breast reconstruction decision support aid (BRAID) for newly diagnosed BC patients Options: Mastectomy or not, suctal carcinoma in situ or stage 1,2,3 a BC	*N* = 55 Women	Participants completed measures of breast reconstruction knowledge, preparation to decide, DCS, anxiety, and BR intentions.	Before randomisation and 2 weeks later.	BRAID is a menu-driven program organised into 10 modules.	BRAID participants returned less surveys. Both interventions increased breast reconstruction knowledge. Both had a significant reduction in DCS. There were no differences. Outcomes: Decision making Patient outcome: affective-cognitive outcomes: knowledge, decisional conflict, empowerment	
Hawley et al., 2016 [27] (USA)	To develop and evaluate a web-based PtDA to determine if this tool could improve the quality of decisions focused on locoregional BC treatment. Options: Mastectomy, mastectomy with reconstruction, or lumpectomy with radiation.	*N* = 101 Newly diagnosed BC patients. *n* = 51 IG patient PtDA first *n* = 50 CG survey first	Pilot study	Knowledge about: Surgical treatment and breast reconstruction. Patient’s appraisal: Decision Satisfaction Scale and perceived values concordance	Web-based PtDA. 16 scenarios (4 treatment attributes, 2 levels for each one): - Risk of cancer coming back - Need radiation treatment - Conserve natural breast - How breast looks after surgery Best fit treatment provided as feedback.	PtDA improves the quality of decisions raising patients’knowledge about treatments, improving appraisal of the process of decision making. Options: Decision making Patient outcome: affective-cognitive outcome, knowledge, satisfaction Behavioural outcomes: match between preferred option and decision made	2006 IPDAS Checklist (64 items)
Durand et al., 2016 [28] (USA)	To develop and test the usability, acceptability, and accessibility of a pictorial encounter decision aid. Targeted at women of low socioeconomic status diagnosed with early-stage BC. Options: Lumpectomy and mastectomy	*N* = 71 P1: *n* = 18 academics and clinicians P2: *n* = 53 people recruited in 3 rounds: - R1: *n* = 22 participants - R2: *n* = 8 participants R3 *n* = 23 P3: *n* = 10 women recruited	Qualitative study with a community-based participatory research approach with 3 phases: P1: prototype development and initial testing P2: iterative prototype testing in undeserved community settings P3: final prototype (Picture Option Grid) testing with target users.	P1: feedback about prototypes P2: think-aloud interviews P3: 9 open-ended questions, examining women’s reactions to the pictorial encounter decision aid.	The pictorial encounter PtDA was derived from an evidence-based table comparing treatment options for BC It uses the same evidence, tabular format, and integrates images that illustrate each answer to nine frequently asked questions.	P1: Researchers and clinicians preferred the black and white prototype. P2: Participants preferred the Picture Option Grid P3: Involving iterative design and testing cycles with multiple stakeholders maximised the usability and acceptability of the intervention, to develop a new and acceptable prototype. Outcomes: Observer reported outcomes, SDM Patient outcome: knowledge, satisfaction	2009 IPDAi Assessment (47 items) URL http://www.optiongrid.com/ (accessed on 24 January 2025)
Serpico et al., 2016 [29] (USA)	To prove if providing accurate info. about BC with a BC Video before initial consultation will decrease distress and increase self-reported knowledge Additional aim: to provide info. to better define processes of adopting SDM in clinical practice Options: Surgical treatment options, lumpectomy, and other surgical procedures	*N* = 156 Patients *n* = 69 G1 subjects (pre survey + BC Video + post survey) *n* = 87 G2 subjects (in clinic survey before surgeon’s meeting)	Prospective observational study composed of two groups.	G1: self-reported of BC knowledge. Perceived distress related to diagnosis. G2: video helpfulness.	The BC Video consists of a standardised overview of BC and general BC surgical treatments	G1 demonstrated an improvement of self-perceived knowledge and patients’ distress decreased overall but not markedly. G2 revealed that patients often seek information in more than one setting. Patients reported the video to be beneficial to their basic understanding of their disease. Distress decreased after the BC Video. Outcomes: Decision making Patient-reported outcome: affective-cognitive outcomes: knowledge, decisional conflict self-efficacy Health outcomes: anxiety, stress	2005 IPDAS (80 criteria/items in 12 broad criteria)
Osaka et al., 2017 [30] (Japan)	To develop a PtDA with patient narratives and determine whether it is more effective than one without patient narratives for women with BC early stage Options: Breast-conserving therapy plus radiotherapy, mastectomy, mastectomy plus breast reconstruction	*N* = 210 *n* = 70 PtDA with patient narratives *n* = 70 PtDA without patient narratives *n* = 70 CG	Single-centre three-arm parallel randomised controlled trial.	DCS and anxiety (STAI) at: T1 (baseline), T2 (post intervention), T3 (1 month after). Satisfaction with DM (effective DM (subscale of the DCS); at: T2, T3. M Demographic and clinical variables.	PtDA comprises Four components: 1. An introduction. 2. A description of surgery for BC (Breast-conserving therapy, Modified Radical Mastectomy, or the latter one plus breast reconstruction, including the probabilities of benefits and harms. 3. facilitation of clarification of values. 4. Guidance in the steps of DM (subscale of the DCS).	PtDA with and without patient narratives are equivalently effective at reducing postoperative DCS in Japanese women with early-stage BC. Outcomes: Decision making Patient outcomes: affective cognitive outcomes, knowledge, decisional conflict, and decisional regret	2005 IPDAS (80 criteria/items in 12 broad criteria) and 2006 IPDAS Checklist (64 items)
Berger-Höger et al., 2017 [31] (Germany)	To develop and pilot a new approach: an inter-professional Informed SDM programme for specialised nurses and physicians to enable them to provide Informed SDM in BC centres. Options: Breast-conserving surgery with radiation, mastectomy, watchful waiting, breast-conserving surgery without radiation	*N* = 34 *n* = 27 BC patients to test the PtDA *n* = 7 BC patients to test the entire intervention Oncologic and BC nurses and physicians previously trained (a programme and a workshop, respectively) Intervention with 3 components: a PtDA for BC (ductal type), a decision coaching led by specialised nurses, structured physician encounters	Mixed methods pilot study: focus groups, individual interviews, and observations.	The acceptance of the intervention by patients and professionals, the applicability to the BC centres’ procedures, patients’ knowledge, patient involvement in treatment SDM assessed with the MAPPIN’ SDM observer instrument MAPPIN’Odyad, barriers. Questionnaires. Structured verbal and written feedback. Video recordings.	Patients attained adequate knowledge (answers: 9–11 of 11). A basic level of patient involvement in treatment SDM was observed for nurses and patient–nurse dyads (M_indicator_(MAPPIN-O_dyad_): 2.15 and M _indicator_ (MAPPIN-O_nurse_): 1.90). Barriers: Physicians barely tolerated women’s preferences not in line with the medical recommendation. Classifying women as inappropriate due to age or education led physicians to neglect eligible women.	SDM coaching is feasible. There are indications structural changes are needed for long-term implementation. Physicians are part of the problem on applying SDM in BC. Outcomes: SDM; nurses and doctors	2009 IPDAi Assessment (47 items)
Savelberg et al., 2017 [32] (The Netherlands)	To develop, alpha test, and improve a patient PtDA for early-stage BC. Additional aim: ensure relevance, usability, comprehensibility, and acceptability of the tool Options: Breast-conserving therapy with radiation therapy and mastectomy with or without radiation therapy	*N* = 26 Professionals (oncologic surgeons, radiation oncologists, medical oncologists, and nurses)	Qualitative descriptive study.	Face-to-face think-aloud interviews, a focus group, and semi-structured telephone interviews. Alpha testing: comprehensibility (patients). Usability (patients and professionals). Acceptability (professionals)	Website with interactive elements to tailor information. Homepage enables personalising the patient PtDA (by a prescription pad from clinician). PtDA: treatment options, pros and cons, side effects, value elicitation statements.	PtDA developed in four iterative test rounds. PtDA well appreciated by professionals and patients, but its acceptability should be proved in practice. Outcomes: SDM	2006 IPDAS Checklist (64 items) Beta testing the PtDA
Hawley et al., 2018 [33] (USA)	To determine the effect of a PtDA (iCanDecide,) regarding locoregional BC treatment and on patient appraisal of SDM. Options: Mastectomy and breast conservation therapy (lumpectomy).	*N* = 537 Women T1: *n* = 248 IG: iCanDecide interactive *n* = 270 CG: iCanDecide static T2: *n* = 245 IG *n* = 251 CG	Randomised controlled trial of newly diagnosed patients with early-stage BC.	T1: Baseline survey T2: (4/5 weeks after) Follow-up survey: primary outcomes (knowledge and values, concordant treatment). Secondary outcomes (decision preparation, deliberation and SDQ)	Website which included: - knowledge-building module, delivered information about key content areas. - values- clarification and feedback exercise about four key attributes of treatment - patient activation module: tailored testimonial.	IG: ↑ odds of making a high-quality decision and higher decision preparation Most patients in both arms made values-concordant treatment decisions. To be effective, patient-facing decision tools should be integrated into the clinical workflow to improve decision making. Outcomes: SDM, Patient Outcome: Affective outcome, knowledge, self-efficacy	Based on IPDAS with no score.
Stankowski-Drengler et al., 2019 [34] (USA)	To examine the impact of a web-based PtDA vs. high-quality websites on patients’ perceptions of information conveyed during the BC surgical consultation, and satisfaction with the DM process.Options:Breast-conserving therapy, mastectomy.	Pre-Survey *N* = 244 patients *n* = 121 decision aid *n* = 123 Website Post-survey *N* = 201 patients *n* = 102 decision aid *n* = 99 Website post-treatment survey *N* = 142 patients *n* = 77 decision aid *n* = 65 Website	Randomised controlled trial.	Demographic info. Surveys: pre-consultation. Post-consultation. Post- treatment: CPS, SDMPS	The PtDA consisted of didactic information about cancers, as well as reconstruction options. Also included were video clinical vignettes to encourage incorporation of personal values and preferences in DM.	There was no association between randomisation arm and perceptions of information conveyed, being asked surgical preference, or satisfaction with the decision process. Surgeon was not associated with satisfaction. Outcomes: SDM Observer reported outcomes Patient outcome: satisfaction with surgeon, not with PtDA	
Ehrbar et al., 2019 [35] (Switzerland)	Main aim: To find out if an online PtDA about fertility preservation, plus counselling, reduces DCS compared to counselling alone. Secondary aims: If knowledge about fertility preservation options, attitude and willingness for fertility preservation and decision regret, as well as satisfaction with the PtDA impact in BC (53%). Options: FP procedures (e.g., egg/embryo freezing) among other treatment options for the BC for fertile women	*N* = 79 Patients. IG *n* = 40 CG: *n* = 39 T1: *n* = 51 *n* = 24 IG *n* = 27 CG T2: *n* = 41 *n* = 18 IG *n* = 23 CG T3: n= 37 *n* = 17 IG *n* = 20 CG	Randomised controlled trial (with a block randomisation) include Ehrbar 2019 BC female patients who were referred by their treating oncologist to fertility preservation counselling IG: link to the PtDA + questionnaire.	(T1: after counselling, T2: 1 month, T3: 12 months) CG: questionnaire (T1–T3) Questionnaire: Knowledge of fertility preservation options, attitude regarding fertility preservation, DCS, DRS, satisfaction with the online decision aid. Final decision about fertility preservation Satisfaction with the decision aid: (IG).	Specific information on cancer treatment, impact on fertility, fertility preservation procedures. Interactive decision-making part with clarification exercises about fertility preservation options.	All participants showed low DCS scores. T1, T2: IG showed a significantly lower total score on DCS than CG. T3: IG still had a lower score in DCS but no longer significant. Outcomes: SDM, Patient outcomes: affective-cognitive, decisional conflict	URL: clinicaltrials.gov (no. NCT02404883)
Berger-Höger et al., 2019 [36] (Germany)	To investigate if an informed SDM intervention for women with BC ductal carcinoma in situ comprising an evidence-based PtDA with nurse-led decision coaching enhances the extent of the SDM behaviour of patients and professionals regarding treatment options, plus barriers Options: Breast-conserving therapy with radiation, mastectomy, watchful waiting (active surveillance), and breast-conserving therapy without radiation	*N* = 192 Women *N* = 16 Centres	Cluster randomised controlled trial with accompanying process evaluation.	The acceptance of the intervention by women and professionals, the applicability to the breast care centres’ procedures, women’s knowledge, patient involvement in treatment decision making assessed with the MAPPIN’ SDM-observer instrument MAPPIN’O_dyad_, and barriers to and facilitators of the implementation were taken into consideration	Treatment Decision Making assessed with the MAPPIN’ SDM observer instrument MAPPIN’O_dyad_, and barriers.	Patients attained adequate knowledge (range of correct answers: 9–11 of 11). A basic level of patient involvement in TDM was observed for nurses and patient–nurse dyads (M_indicator_(MAPPIN-O_dyad_): 2.15 and M(MAPPIN-O_nurse_): 1.90). Relevant barriers were identified, physicians barely tolerated women’s preferences that were not in line with the medical recommendation. Classifying women as inappropriate for Informed SDM due to age or education led physicians to neglect eligible women during the recruitment phase. Outcomes: SDM Cost and cost analyses Patient outcomes: affective, knowledge, patient clinical communication Behavioural match: between preferred and level of participation Process outcomes: consultation length	2009 IPDAi Assessment (47 items)
Lee et al., 2019 [37] (Taiwan)	To describe the developmental process of creating an animated comic as a web-based surgery patient PtDA for patients with BC Options: Lumpectomy breast-conserving surgery or mastectomy	Action phase *N* = 11 BC patients Evaluation phase *n* = 1 BC surgeon *n* = 7 BC survivors	Mixed methods	Planning phase: web-based personal stories. Action phase: semi-structured interviews. Evaluation Phase: Surgeon comment, feedback from a focus group	Web-based animated comic with audio explanations. It contains 8 chapters: 1. Appearance of a lump. 2. Diagnosis. 3. Uncertainty waiting. 4. Fear. 5. Choosing life. 6. Treatment. 7. Type of surgery. 8. Being reborn.	Comic acts as an information resource and is aimed at patients’ understanding of impacts of emotions arising when suffering from BC. Therapeutic tool that facilitates self-reflection and self-healing among newly diagnosed patients. Outcomes: Patient-reported outcome Health-related quality of life Patient outcome: affective-cognitive outcomes Health outcomes: anxiety, depression, stress	
Olling et al., 2019 [38] (Denmark)	To explore if a PtDA improved SDM and supported a patient-centred approach in BC and lung cancerOptions: Accept or decline therapy adjuvant after surgery	*N* = 54 Phase 1: *n* = 29 no PtDA. Phase 2: *n* = 25 patient PtDA Clinicians’ recruitment: *n* = 3 nurses	Nonexperimental, observational study. Cohort study. Phase 1: baseline cohort. Phase 2: intervention cohort.	Real-life observations using OPTION 12. A nurse made and rated the observation. Another nurse listened and rated the recording. All the nurses took turns at the different tasks.	Using a PtDA increased the OPTION score. The same results in BC and LC. The same results without patient PtDA and with PtDA.	PtDA improved SDM behaviour and promoted a patient-centred approach. PtDA increased the overall OPTION score. PtDA supports SDM in consultations independently of type of decision and department. Outcomes: SDM Observer-reported outcomes Patient outcomes: satisfaction with decision making	2009 IPDAi Assessment (47 items)
Savelberg et al., 2019 [39] (The Netherlands)	To explore the experiences, issues, and concerns of early-adopter professionals with regards to SDM in BC Options: Breast-conserving therapy or mastectomy	*N* = 27 Clinicians’ recruitment: *n* = 9 BC surgeons *n* = 11 nurse practitioners *n* = 7 nurses	Qualitative descriptive study. Face-to-face interview.	Topics: SDM attitude and behaviour. Knowledge. patient PtDA use	Patients access with login code. In the first attempt, they mark their treatment options. Patient PtDA includes a video to propitiate the conversation about SDM.	Most clinicians focused only on the first steps of SDM. The other steps were regarded as challenging. Surgeons delegating responsibility to nurses. Clinicians unaware of their lack of competency about SDM. Clinicians require training on SDM, willing to use the approach by surgeons, and test their skills before PtDA implementation. Outcomes: SDM Observed reported outcomes	
Søndergaard et al., 2020 [40] (Denmark)	To evaluate the impact on BC consultation length and decisions made when practicing SDM with the use of an in-consult PtDA. Options: Adjuvant treatment after early-stage BC: hemotherapy, human epidermal growth factor receptor 2 targeted treatment, endocrine treatment, zoledronic acid treatment, and/or adjuvant radiotherapy	*N* = 261 BC patients *n* = 64 CG *n* = 63 IG	Prospective cohort study.	Time registration. OPTION 12. DRS (6 months after).	PtDA design supports a 4-step approach to SDM: choice talk, preference talk, option talk, and decision talk. Patients were prepared with brief information about SDM. PtDA used by the doctor in the consultation and, later, given to the patient.	The introduction of SDM and an in-consult patient PtDA did not increase the consultation length. SDM led to more conservative decisions, although the degree of the impact depended on the clinical situation. Outcomes: Slight increase in the number of patients declining adjuvant treatment for BC. SDM Cost and cost analysis	2013 IPDAS Checklist (44 items).
Lin et al., 2019 [41] (Taiwan)	To develop an app as a PtDA and examine the feasibility and usability of it among women newly diagnosed with BC. Options: Breast reconstruction surgery: implant-based breast reconstruction or autologous (using the patient’s own tissue) breast reconstruction	P1: Development (a software engineer, a surgeon, nurses, an informaticist, and a researcher in BC) P2: Feasibility and usability, *N* = 11 women	2 phases. 1 Prototype design. 2 Pilot quasi-experimental study pre-test and post-test.	Sociodemographic info. DCS. Qualitative questionnaire about acceptability and satisfaction	Pink Journey contains information about surgical options, including breast reconstruction and mastectomy, advantages and disadvantages, the complication probability, a value clarification exercise for the patient’s self-evaluation, and a summary of the participants’ SDM process.	Less decisional conflict in I on each subscale of the DCS Most women felt the app was both helpful and user-friendly. The app increased their participation in SDM, helped them obtain more accurate risk perceptions, and clarified their values. It also helped the women make decisions regarding breast reconstruction more confidently. Outcomes: SDM Health-related quality of life Patient outcomes: affective-cognitive outcomes, knowledge, satisfaction, decisional conflict Behaviour outcomes: match between preferred option and decision made	
Fang et al., 2021 [42] (Taiwan)	To examine the effects of a decision support app on SDM quality and psychological morbidity for women considering breast reconstruction surgery due to BC. Options: Breast reconstruction options: implant-based or autologous reconstruction. Surgical techniques: Traditional or endoscope-assisted. Timing of reconstruction: Immediate or delayed	*N* = 96 CG: *n* = 48 pamphlet IG: *n* = 48 pamphlets + app.	Randomised controlled trial with permuted block randomisation.	T0: baseline data collection (demographic and clinical). DCS, subscale involvement in breast reconstruction SDM, process scale, DRS, BIS, HADS at 4 T after surgery: T1: 1 week after. T2: 1 month after. T3: 8 months after. T4: 12 months after.	Participants watch a video compatible with the pamphlet information. They were taught on values clarification exercises to rank their concerns. They were prompted to discuss the opinion with their significant others. The SDM document is printed.	Body image distress declined in IG and increased in CG. There were no differences in DCS, decision regret, anxiety and depression between IG and CG. Outcomes: SDM Patients reported outcomes Patient outcomes: affective-cognitive outcomes, satisfaction Behavioural outcomes: match between preferred and decision made.	
Burton et al., 2021 [43] (UK)	To improve treatment through SDM for older women with BC (which is a high-risk population group) by developing and testing two decision support interventions, each one supporting one option/decision. Options: Primary endocrine therapy or surgery with adjuvant therapies or to have adjuvant chemotherapy after surgery or not	*N* = 82 Women (>70 years)	Multi-centre, parallel group, pragmatic, cluster randomised controlled trial, DCS nested within a larger cohort study of older women with early BC.	Primary outcome: improvement in QoL.	The decision support interventions were developed to ensure that the information They contained was accurate, relevant, and desired by this population. Each decision support intervention had three components: (1) an online risk prediction model, (2) a brief PtDA, and (3) an information booklet	Reach: The online tool was accessed on 324 occasions by 27 clinicians. Reasons for non-use: the patient had decided or there was no online access in the clinic. Of the 32 women, 15 from the IG and 6 CG were offered a choice of treatment. Fidelity: Clinicians used the online tool in different ways, during the consultation or checking the online survival estimates before the consultation. Adaptation: Evidence when using the decision support interventions. Barriers: A lack of infrastructure for the use of the tool. The brief PtDA was rarely used. Mediators: SDM, most patients felt able to contribute to decision making and expressed high levels of satisfaction with the process. Result: 6 patients reported the PtDA to be very useful, 1 somewhat useful, and 2 moderately useful. Outcomes: Increase SDM Observer reported outcomes of the use of app	
Raphael et al., 2021 [44] (The Netherlands)	Main aim: To assess in BC if PtDA increases decisional quality, perceived SDM level, and knowledge on the options. Additional aim: To observe if PtDA impacts on the choice of radiation treatment level and on consultation length. Options: Radiation therapy or not.	*N* = 403 BC patients *n* = 214 CG no PtDA *n* = 189 IG patient PtDA Patients’ recruitment: 13/19 radiation treatment centres 2017–18 Clinicians’ recruitment: *n* = 33 Surgery department *n* = 133 radiation treatment department	Multi-centre pre- and post- intervention study. Patients: T1: 3 days after decision T2: 3 months after final treatment decision about radiation treatment. Clinicians: case report form, tumour, treatment, consultation length.	Tests, DCS, SDM—Q9, CollaboRATE: Knowledge on patient PtDA options, preferences on SDM attributes, final treatment decision	Online patient PtDA starts with an introduction on SDM. Explanations about how radiation treatment is performed (in text and in animation film). Information on the possible effects and side effects of radiation treatment. It elicits a patient’s preferences.	Corrections in age and educational level. No differences in DCSnor perceived SDM. IG less additional treatment and had more knowledge. Attributes: recurrence risk, clinician’s advice, choose radiation treatment, give peace. No increase in consultation time. Outcomes: SDM Patient outcomes: affective cognitive outcomes, knowledge, no changes in decisional conflict	2006 IPDAS Checklist (64 items PtDA score (IPDAS): 83/100 URL: https://beslissamen.nl/ (in Dutch) (accessed on 24 January 2025).
Schubbe et al., 2021 [45] (USA)	To explore strategies that promote the BC conversation aids sustained use and dissemination. Additional aim: To evaluate differences between two conversation aids (text-based vs. picture-based) considering varied socioeconomic strata. Options: Mastectomy and breast-conserving surgery with radiation	*N* = 43 Patients’ recruitment: *n* = 18 Option Grid *n* = 25 Picture Option Grid *N* = 16 Surgeons’ recruitment: *n* = 5 Option Grid *n* = 6 Picture Option Grid *n* = 5 usual care *N* = 14 Stakeholders’ recruitment: 3 nurse practitioners, 3 nurses, 1 physician assistant, 1 social worker, 6 nonclinical	Multi-site randomised controlled trial.	Normalisation Process Theory: coherence. Cognitive participation. Collective action. Reflexive monitoring.	Option Grid: evidence-based information on breast-conserving surgery with radiation treatment and mastectomy in a comparative table. Picture Option Grid: same information with fewer words and pictures.	Patients and surgeons felt the conversation aids should be used in BC care in the future. Patients: ↑ Socioeconomic Status: conversation aids influenced more treatment discussion. ↓ Socioeconomic Status: conversation aids influenced more decision making. Conversation aids did not lengthen consultation time. Outcomes: SDM Cost and cost analysis Patient outcomes: satisfaction, patient clinical communication. Process outcomes: no increase in consultation length	
Wyld et al., 2021 [46] (UK)	Main aim: To evaluate in older women with BC the decision support interventions effects on QoL. Additional aim: The same evaluation with survival, decision quality (coping and decision regret), and two treatments. Options: (1) surgery plus adjuvant endocrine therapy vs. primary endocrine therapy (2) adjuvant chemotherapy vs. no chemotherapy	46 BC units participate. *N* = 1339 women (≥70 years). CG: *n* = 669 IG: *n* = 670	Multicentre, parallel group. Cluster randomised controlled trial (CONSORT guidelines). Randomisation stratified by primary endocrine therapy and chemotherapy rates to avoid bias.	Baseline, 6 weeks, 6 months. QoL: EORTC QLQ-C30, QLQ-BR23, QLQ-ELD14 SDM: CollaboRATE, DRS (5 items) Psychology: STAI, BIPQ, Brief COPE Other data: Age, Charlson Comorbidity Index score 36, ADL, Instrumental ADL MMSE, bridged Patient, Generated Subjective Global Assessment, tumour stage/grade/biotype and treatment. Clinicians: Tool evaluated using registration system (n and duration of logins at each site). Info on use of the decision support interventions from the case report form about the consultation.	Decision support intervention; online decision algorithm, booklets, brief PtDA to inform choices between treatment options. Decision support intervention adjusted for co-morbidities and frailty. The tool produces personalised survival outcomes according to fitness, frailty, stage, treatment choice, disease biology. Tool developed and used the preferred informational content, format, terminology, and media for this population and were piloted extensively in this age group.	No significant difference in global QoL at 6 months. When offered a choice of primary endocrine therapy vs. surgery plus endocrine therapy, greater knowledge in IG. Treatment choice was altered, among those with oestrogen receptor-positive disease; more choose primary endocrine therapy in IG than CG. A high quality of SDM in both arms. Psychological results were no different among groups. Survival similar in both arms. Outcomes: SDM Patient outcomes: affective-cognitive outcomes: knowledge Health outcomes: quality of life, no changes. Improved decision quality	URL: Age Gap Decision ToolVC; https://agegap.shef.ac.uk/ (accessed on 24 January 2025).
van Strien-Knippenberg et al., 2021 [47] (The Netherlands)	To design decision-relevant information about adjuvant BC treatment in cocreation with patients, suiting their needs and easily understandable. Options: Adjuvant treatments after surgery: chemotherapy, hormone therapy, or targeted therapies	P1(3 sessions): *N* = 10, *N* = 8 and *N* = 7 patients P2: *N* = 10 patients + *N* = 10 healthcare providers	Qualitive approach in 2 phases: (1) cocreation, (2) user testing.	Demographics and test for educational level. P1: Important decision timeline, relevant info. DM, health literacy, self-reported questionnaire about nausea and fatigue. P2: questions about the PtDA, questions about the visualisation.	PtDA with personalised estimates information: summary table about benefit/harm treatment options. Survival rates. Side effects.	Important needs: 1. Overview after the diagnosis, 2. Clear benefit/harm info. about treatment options. Important value clarification method resume. Bar graph is the most appropriate way for survival rates. The concept of personalisation was not understood. Outcomes: Observer and patient reports SDM Patient outcome: affective-cognitive outcomes, knowledge Behavioural outcomes: match between preference and decision made or self-efficacy	2021 IPDAS 2.0. Checklist (11 core domains)
Ter Stege et al., 2021 [48] (The Netherlands)	To develop a patient PtDA that could support patients with BC in making an informed decision Options: Immediate or delayed reconstruction, a different type, such as implant-based or autologous reconstruction	*N* = 86 S1: *n* = 16 experts S2: *n* = 17 patients, *n* = 33 healthcare professionals S4: *n* = 6 patients, *n* = 7 healthcare professionals, *n* = 7 representatives of BC organisation	Development in 4 stages: 1. Multidisciplinary team, 2. Needs assessment, 3. Creation, 4. Acceptability and usability.	Semi-structured interviews (patients) and survey (healthcare professionals). S4: think-aloud (patients) and interviews (healthcare professionals and representatives).	1. A consultation sheet, surgeons to introduce the choice. 2. An online tool including an overview of reconstructive options, the pros and cons, information on the consequences, exercises to clarify values, and patient stories. 3. A summary sheet with patients’ values, preferences, and questions to help inform and guide the discussion between the patient and her plastic surgeon.	The PtDA was perceived to be informative, helpful, and easy to use by patients and healthcare professionals. Patients prioritise cure over aesthetics when deciding on immediate breast reconstruction. They seek tailored, objective information about breast reconstruction options, outcomes, and recovery. Healthcare professionals recognised the need for a PtDA. Outcomes: SDM Observed a patient-reported outcome Patient outcomes: knowledge, satisfaction.	2009 IPDAi Assessment (47 items)
Pan et al., 2024 [49] (China).	Explore the perceptions and needs of BC patients regarding the utilisation of web-based surgical decision aids. Options: Mastectomy and breast-conserving surgery	*N* = 16 BC patients.	Descriptive qualitative study. The study used a thematic analysis to explore the perceptions and needs of BC patients regarding the use of surgical PtDA.	Semi-structured interviews with purposive sampling that were audio-recorded and transcribed verbatim. A thematic analysis was conducted using NVivo 12 software.	Criteria for Reporting Qualitative Research (COREQ) checklist. A guide included a mix of six open-ended questions regarding the perspectives and needs in the decision-making phase that impact the use of PtDA.	Themes with corresponding sub-themes: (1) informative and useful content (need to know as much information as possible, easy to understand, and presented in multiple ways and highly credible from a reliable resource); (2) user-friendly on design (easy to operate, simple function, and man–machine interaction); (3) suggested timing of use. Outcomes: SDM Patient reported outcomes	2013 IPDAS Checklist (44 items)

Note: ADL: activities of daily living; BC: breast cancer; BIPQ: Brief Illness Perceptions Questionnaire; BIS; The Body Image Scale; Brief COPE: the short version of the Coping Orientation to Problems Experienced Inventory; CG: Control Group; CollaboRATE: a short scale for Shared Decision Making to patients, parents, or their representatives; CPS: Control Preferences Scale; DCS: Decisional Conflict Scale; DM: Decision Making (subscale of the DCS); DRS: Decision Regret Scale; eHEALS: e-Health Literacy Scale; EORTC: European Organisation for the Research and Treatment of Cancer; EORTC QLQ-BR-23: European Organisation for Research and Treatment of Cancer Breast with 23 items of cancer-specific supplement information; EORTC QLQ BR-45: European Organisation for Research and Treatment of Cancer of Cancer Breast—Breast-Cancer-Specific Questionnaire; EORTC QLQ-C-30: European Organisation for Research and Treatment of Cancer Breast—Cancer-Specific Quality of Life Questionnaire—Core Questionnaire; EORTC QLQ-ELD-14: European Organisation for the Research and Treatment of Cancer Quality of Life Elderly Cancer Patients; IG: Intervention Group; G: Group; HADS: The Hospital Anxiety and Depression Scale; IPDAS: International Patient Decision Aid Standards; MAPPIN’SDM: extent of informed shared decision making assessed by the observer-based instrument MAPPIN-Odyad of the validated inventory Multifocal Approach to the sharing‘ IN Shared Decision-Making; N: sample size; OPTION: the observing patient involvement scale; P: phase; PtDA: Patient Decision Aid; QoL: quality of life; SDM: shared decision making; S: sample; SDQ: Subjective Decision Quality scale; SDMPS: Satisfaction with the Decision-Making Process Scale; STAI: Spielberger Short State-Trait Anxiety Inventory; R: round; T: time point.

**Table 2:** Risk of bias according to JBI, the critical assessment tool to cross-sectional studies.

Study	Q1	Q2	Q3	Q4	Q5	Q6	Q7	Q8
Garverlink et al., 2013 [21]	✔	✔	✔	✔	✔	✔	✔	✔
Lam et al., 2013 [22]	✔	✔	✔	✔	✔	✔	✔	✔
Shaffer et al., 2013 [23]	✔	✔	✔	✔	✔	✔	✔	✔
Shaffer et al., 2013 [24]	✔	✔	✔	✔	✔	✔	✔	✔
Shaffer et al., 2014 [25]	✔	✔	✔	✔	✔	✔	✔	✔
Manne et al., 2015 [26]	✔	✔	✔	✔	✔	✔	✔	✔
Hawley et al., 2016 [27]	✔	✔	✔	✔	✔	✔	✔	✔
Durand et al., 2016 [28]	✔	✔	✔	✔	✔	✔	✔	**∕**
Serpico et al., 2016 [29]	✔	✔	✔	✔	✔	✔	✔	✔
Osaka et al., 2017 [30]	✔	✔	✔	✔	✔	✔	✔	✔
Berger-Höger et al., 2017 [31]	✔	✔	✔	✔	✔	✔	✔	±
Savelberg et al., 2017 [32]	✔	✔	✔	✔	✔	✔	✔	**∕**
Hawley et al., 2018 [33]	✔	✔	✔	✔	✔	✔	✔	✔
Stankowski-Drengler et al., 2019 [34]	✔	✔	✔	✔	✔	✔	✔	✔
Ehrbar et al., 2019 [35]	✔	✔	✔	✔	✔	✔	✔	✔
Berger-Höger et al. [36]	✔	✔	✔	✔	✔	✔	✔	✔
Lee et al., 2019 [37]	✔	✔	✔	✔	±	✘	✔	**∕**
Olling et al., 2019 [38]	✔	✔	✔	✔	±	✘	✔	✔
Savelberg et al., 2019 [39]	✔	✔	✔	✔	±	✘	✔	**∕**
Søndergaard et al., 2020 [40]	✔	✔	✔	✔	±	✘	✔	✔
Lin et al., 2019 [41]	✔	✔	✔	✔	✔	±	✔	✔
Fang et al., 2021 [42]	✔	✔	✔	✔	✔	✔	✔	✔
Burton et al., 2021 [43]	✔	✔	✔	✔	✔	✔	✔	✔
Raphael et al., 2021 [44]	✔	✔	✔	✔	✔	✔	✔	✔
Schubbe et al., 2021 [45]	✔	✔	✔	✔	✔	✔	✔	✔
Wyld et al., 2021 [46]	✔	✔	✔	✔	✔	✔	✔	✔
van Strien-Knippenberg et al., 2021 [47]	✔	✔	**∕**	**∕**	±	**∕**	✔	**∕**
Ter Stege et al., 2021 [48]	✔	✔	**∕**	**∕**	±	**∕**	✔	**∕**
Pan et al., 2024 [49]	✔	✔	**∕**	**∕**	±	±	✔	**∕**

Notes: ✔ Yes, ✘ No; ± Partially applicable; **∕** Not applicable; Q1: Were the criteria for inclusion in the sample clearly defined? Q2: Were the study subjects and the setting described in detail? Q3: Was the exposure measured in a valid and reliable way? Q4: Were objective, standard criteria used for measurement of the condition? Q5: Were confounding factors identified? Q6: Were strategies to deal with confounding factors stated? Q7: Were the outcomes measured in a valid and reliable way? Q8: Was appropriate statistical analysis used?

## Abbreviations

The following abbreviations are used in this manuscript:

SDM	Shared decision making
PtDA	Patient decision aid
BC	Breast cancer
IPDAS	International Patient Decision Aid Standards
PRISMA	Preferred Reporting Items for Systematic reviews and Meta-Analyses
